# Development of a screening method for determining sodium intake based on the Dietary Reference Intakes for Japanese, 2020: A cross-sectional analysis of the National Health and Nutrition Survey, Japan

**DOI:** 10.1371/journal.pone.0235749

**Published:** 2020-09-15

**Authors:** Chika Okada, Hidemi Takimoto

**Affiliations:** Department of Nutritional Epidemiology and Shokuiku, National Institute of Health and Nutrition, National Institutes of Biomedical Innovation, Health and Nutrition, Tokyo, Japan; International University of Health and Welfare, School of Medicine, JAPAN

## Abstract

**Background:**

Although assessing nutrient intake through dietary surveys is desirable, it can be effort- and time-intensive. We aimed to develop a brief screening method for determining sodium intake in order to raise public awareness regarding the Dietary Reference Intakes for Japanese (DRI-J) 2020.

**Methods:**

Using data from the 2015 National Health and Nutrition Survey, we compared dietary behaviours obtained from a self-administered questionnaire according to sodium intake, which was assessed from one-day dietary records by a semi-weighed method. Participants were divided into 4 groups based on the reference values of sodium (salt equivalent) shown in the DRI-J. We also randomly divided the participants into development and validation groups, and used logistic regression analysis to identify predictive factors for sex-specific DRI-J (<7.5 g/day in men and <6.5 g/day in women) and above-average intakes (≥10 g/day in men and women).

**Results:**

Among the 6,172 Japanese individuals aged ≥20 years old, participants with lower sodium intake were found to use nutrition labels and had a lower frequency of eating out than those with higher intakes (*P* for difference < .001). Our final model for predicting sodium intake included adjusted sex, age, dietary behaviours, and consumption of mainly processed foods. In the development group, areas under the receiver operating characteristics curves were 0.747 and 0.741 for adherence to sex-specific DRI-J and above-average intake, respectively. The corresponding values in the validation group were 0.734 and 0.730, respectively.

**Conclusions:**

This method could easily identify sodium intake using dietary behaviours and specific food consumption, and is expected to be widely useful for health and nutrition education in Japan.

## Introduction

The Government of Japan has published the revised Dietary Reference Intakes for Japanese in the 2020 edition (hereinafter referred to as DRI-J 2020), which will be applicable for 5 years, starting from the 2020 fiscal year [1]. For the maintenance and promotion of public health and prevention of lifestyle-related diseases, the DRI-J 2020 committees and working groups conducted literature reviews based on evidence and revised the reference values of energy and nutrient intake in healthy individuals and populations. Target subjects of the DRI-J 2020 included individuals and populations who were able to lead independent daily lives, despite having risk factors for lifestyle-related diseases or frailty (in older individuals).

One of the notable points of the DRI-J 2020 is that the reference value of sodium intake, denoted as salt equivalent and defined as “the tentative dietary goal for preventing lifestyle-related disease(DG)”, which was set to less than 7.5 g per day and 6.5 g per day for men and women, respectively; this was 0.5 g lower than in the previous edition [1, 2]. Sodium intake in Asia is known to among the highest in the world [3]. Therefore, in order to reduce the incidence and aggravation of lifestyle-related diseases, the present Japanese population should try to achieve the current target intake through the Plan-Do-Check-Act(PDCA) cycle [1]. Considering feasibility, the reference value of salt is the immediate target, and has been set at the intermediate value of 5 g/day in the WHO guidelines and the sex-specific intake of the National Health and Nutrition Survey, 2016 [4, 5]. Ideally, it is desirable to assess the intake of energy and nutrients through a dietary survey, however, this is considerably difficult to practice as it requires time and effort, particularly among individuals who have no interest in nutrition. Therefore, an easy method for checking their salt intakes in a short time is required.

We aimed to develop a screening method that makes it possible to assess sodium intake using a simple questionnaire on dietary behaviours and specific food consumptions, based on previous studies [6–8], in order to raise public awareness on the target value and to promote salt reduction in Japanese.

## Materials and methods

### The National Health and Nutrition Survey

This study was conducted using data from the National Health and Nutrition Survey (NHNS) 2015 approved by the Ministry of Health, Labour and Welfare. Therefore, the study was exempted from the Institutional Review Board approval and the requirement for informed consent, owing to secondary use of anonymous data.

The NHNS was conducted in 2015 among residents of 300 randomly selected areas with public health centres, across all prefectures in Japan. It had been designed to include a physical examination questionnaire, a dietary questionnaire, and a lifestyle questionnaire (for subjects aged 20 years or older, including questions on dietary behaviour, physical activity, sleep pattern, smoking, drinking, and dental hygiene).

The subjects in our study were limited to 6,172 individuals (2,840 men and 3,332 women) aged 20 years or older who responded to both the lifestyle and dietary questionnaire. For developing the screening method, we used complete-case analysis to handle the missing data.

### Assessment of sodium intake

The details of the dietary survey protocol are reported elsewhere [9]. In brief, the survey was performed on a usual eating day, except for Sundays, holidays, or other special days on which dietary patterns could change. Participants recorded their dietary status for each meal, dish name, food name, volume, waste volume, and proportional distribution among each household member using a semi-weighed method. After getting instructions, several trained dieticians checked their dietary records and the nutrient intakes were calculated based on the 2010 Standard Tables of Food Composition in Japan [10]. Food intake was classified into groups of 17 large (e.g. cereals, vegetables, and fish and shellfish), 33 medium (e.g. rice and rice products, wheat flour and wheat products, green and yellow vegetables, other vegetables, pickled vegetables, raw fish, shellfish, seafood, and processed products), and 98 small classifications (e.g. rice, bread, instant noodles, tomatoes, carrots, horse mackerels and sardines, and fish products-salted, half-dried, and dried) based on the food group tables in the NHNS.

Food group intakes was divided into four categories (none, ≤1.0 unit, 1.1–3.0 units, and >3.0 units) according to the primary unit for each food (e.g., 1 slice and 1 piece), except for instant noodles (none, ≤0.5 pack, 0.6–1.0 pack, and >1.0 pack) owing to the high salt contents.

Sodium intake was divided into 4 groups based on the salt equivalent in DRI-J 2020 and the average population value. The 4 groups were <6.5 g per day (from the reference value for women) [1], 6.5–7.5 g per day (from the reference value for men) [1], 7.5–10.0 g per day (from average intake of adults 9.9 g per day) [5], and 10.0 g per day and above. For developing the screening method, we defined the adequate intake as “sex-specific DRI-J 2020 (<6.5 g in women and <7.5 g in men)” or “the median of sex-specific DRI-J 2020 (<7.0 g in both sexes)” and the excess intake as “above-average intake” to predict sodium intake.

### Dietary behaviours

Participants filled out the questionnaire, as follows: (1) How often do you refer to the nutrition label when you buy food (always, sometimes, rarely, or never); (2) Which nutritional content you think is necessary in the nutrition label (1-energy; 2-protein; 3-fat; 4-carbohydrates; 5-sodium or salt equivalent; 6-saturated fatty acid; 7-cholesterol; 8-sugar; 9-dietary fibre; 10-vitamins and minerals; 11-other; 12-none (yes or no for all)); (3) The frequency of eating-out (≥2 times/day, 1 time/day, 4–6 times/week, 2–3 times/week, 1 time/week, under 1 time/week or none); (4) The frequency of take-out including lunch boxes and ready-to-eat dishes (same options as eating-out); and (5) The frequency of ideal combination of three dishes containing the staple food (i.e., rice, breads, and noodles), a main dish (i.e. dishes from fishes, meats, eggs, and soybeans), and side dishes (i.e. dishes from vegetables, seaweeds, and mushrooms) more than twice a day (almost everyday, 4–5 days/week, 2–3 days/week, and almost none). We categorised the responses to the utilization of nutrition labels and the frequency of eating-out into binary variables (always/sometimes vs. rarely/never and ≥2 vs ≤1 times/week, respectively) to develop the screening method for high and low sodium intake.

### Statistical analyses

All statistical analyses were performed using the SAS version 9.4 software (SAS Institute Inc., Cary, NC) and *P* values <0.05 were regarded as significant.

A chi-square test was used for the comparison of dietary behaviours between the groups. We combined men and women as the desirable intake of 5 g per day was the same regardless of sex, although the reference values of DRI-J 2020, considering feasibility, would be separate for men and women. We further tested pair-wise contrasts using the Dwass, Steel, Critchlow-Fligner (DSCF) multiple comparison analysis [11].

**Fig 1:**
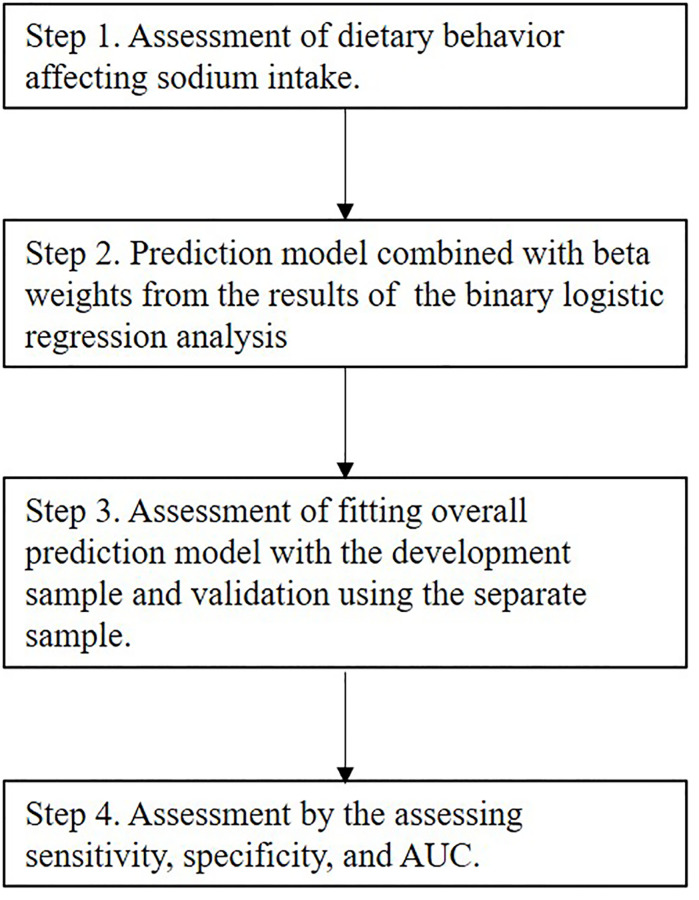
An overview of the analysis steps applied for this study. AUC, areas under receiver operating characteristic curve.

For validation of the method, we randomly divided the subjects into two groups: the development and validation groups, considering area clusters, sex, and 10-year age strata, using random number seed 1234567. We used logistic regression analysis for adequate intake based on DRI-J 2020 (<7.5 g per day or ≥7.5 g per day in men and <6.5 g per day or ≥6.5 g per day in women) and high intake based on average intake (>10 g per day or ≤10 g per day in men and women). The models were re-applied using <7.0 g in both sexes, the median value of sex-specific DRI (salt intake) for adequate intake. The adjusted variables included dietary behaviours and processed food consumption with high contribution of sodium intake (4 portion sizes as intake per day), in addition to sex and age, and a cut-off of *P*-value <0.05 was finally applied for inclusion in the multivariable model. We calculated sensitivity, specificity and the receiver operating characteristics curves (AUC) in the development and validation groups, using the estimated regression coefficients from the final model, and also assessed the predicted probability according to several cut-offs for practical use (Fig 1).

## Results

The characteristics of 6,172 participants according to their sodium intakes are presented in Table 1. Participants with higher sodium intake were more likely to be men, aged 60–69 years, and smokers, and have a BMI ≥25 kg/m^2^ (*P* for difference < .001 for all). Furthermore, the proportion of those eating three meals a day was higher among participants with high sodium intake (*P* for difference < .001).

**Table 1:** Characteristics of participants.

	Sodium (Salt equivalent) intake				
	< 6.5g	6.5-<7.5g	7.5-<10g	≥10g	
No. of participants	1,101	616	1,684	2,771	
Male, %	31.2	34.7	42.3	56.7	<0.001
Age, years, %					
20–29	7.9	8.0	7.4	7.5	<0.001
30–39	14.2	14.1	10.6	10.4	
40–49	19.5	20.8	16.3	15.1	
50–59	13.9	14.0	15.5	16.6	
60–69	15.3	19.5	24.5	24.3	
70–79	15.4	14.6	16.5	19.1	
≥80	13.8	9.1	9.2	7.1	
BMI, kg/m^2^, %					
<18.5	30.2	24.2	25.1	22.4	<0.001
18.5–24.9	54.1	58.3	55.6	55.9	
25.0–29.9	12.9	14.9	16.4	18.5	
≥30	2.8	2.6	2.9	3.2	
Smoker, %	14.4	15.2	15.1	18.8	<0.001
Three meals a day, %	70.6	83.0	85.7	89.3	<0.001

Body mass index (BMI) was calculated as weight (kg) divided by height squared (m^2^) using the physical condition questionnaire. Missing data were excluded in the analysis of BMI and smoking status.

Table 2 shows the distribution of dietary behaviours according to sodium intake in 5,205 participants, who ate three meals a day. In order to avoid confusion with the quantitative effects of the meal itself, participants who skipped any meal were excluded. Participants with low sodium intake were more likely to utilise nutrition labels and had a lower frequency of eating-out (*P* for difference <0.001, respectively), however, the frequency of taking out was not. Significant differences were observed in the needs of food labelling for sodium and carbohydrate among the groups. The multiple comparison analysis (S1 Table) showed that dietary behaviours such as utilisation of nutrition labels, frequency of eating out, and frequency of ideal combination of dishes were significantly different among the sodium intake groups.

**Table 2:** Dietary behaviours according to sodium intakes (salt equivalent).

	Sodium (salt equivalent) intake				*P* for difference
	<6.5 g	6.5-<7.5 g	7.5-<10 g	≥10 g	
No. of participants	777	511	1,443	2,474	
Utilisation of nutrition label, %					
Always	10.5	10.8	9.7	7.9	<0.001
Sometimes	33.6	38.0	34.6	31.4	
Rarely	35.9	30.8	32.6	33.5	
Never	20.0	20.4	23.1	27.2	
Nutrients needed in food label, %					
Energy	45.0	46.9	43.8	42.1	0.16
Protein	24.4	25.9	25.8	25.6	0.89
Fat	30.8	31.9	29.0	27.2	0.08
Carbohydrates	17.6	17.6	14.7	13.8	<0.05
Sodium (as salt equivalent)	30.7	29.1	28.3	25.6	<0.05
Saturated fatty acid	7.4	7.4	7.5	5.6	0.07
Cholesterol	25.1	28.7	26.8	24.7	0.18
Sugar	30.8	27.7	29.5	27.7	0.32
Dietary fibre	29.0	29.5	30.7	27.8	0.27
Vitamins and minerals	29.6	30.7	29.5	29.8	0.96
Others	3.0	3.4	3.2	2.4	0.43
None	26.2	24.6	25.9	28.6	0.12
Frequency of eating-out, %					
≥2 times/day	0.4	0.6	0.7	0.6	<0.001
one time/day	0.5	2.0	2.1	2.7	
4–6 times/week	2.2	3.8	3.1	4.6	
2–3 times/week	5.6	10.0	7.8	9.4	
one time/week	12.6	15.2	14.0	15.5	
under one time/week	46.3	47.9	48.1	44.9	
None	32.3	20.6	24.3	22.3	
Frequency of taking-out, %					
≥2 times/day	0.5	0.8	0.6	0.6	0.78
One time/day	2.2	2.2	3.1	2.8	
4–6 times/week	3.8	3.0	4.0	4.7	
2–3 times/week	17.8	19.8	15.9	16.6	
One time/week	14.1	14.0	14.0	13.7	
under one time/week	33.4	36.5	35.9	35.0	
None	28.2	23.8	26.5	26.6	
Frequency of ideal dietary pattern, %					
Almost everyday	49.7	53.6	55.8	56.8	<0.01
4–5 days/week	17.5	20.6	16.1	17.4	
2–3 days/week	22.9	16.8	20.1	18.4	
Almost none	9.8	9.0	8.0	7.4	

Missing values were excluded in the analysis of each item.

An ideal dietary pattern was one in which the staple food, main dish, and side dish were consumed more than twice a day.

A complete-case analysis included 6,066 participants and the development and validation groups had 3,056 and 3,010 participants, respectively. Our final model included adjusted sex, age, three dietary behaviours (skipping any meal, frequency of eating-out, and ideal combination of dishes), and seven types of processed foods consumed, namely, breads, instant noodles, fish products: salted, half-dried, and dried, and fish paste, meat products: ham and sausage, cheese, and pickled vegetables (Table 3). The final logistic model produced significant overall fit with the dummy variable approach (likelihood ratio <0.0001 for criteria of sex-specific DRI-J (Adequate intake) and above average (Excess intake), respectively). From these results, we used the following equitation to calculate the probability:

$$\begin{array}{l} logit\left(Adequate\;intake\right)=0.4692-0.0788\;X_{male}+\left(Age:0.3792\;X_{20-29y}+0.5221\;X_{30-39y}+0.2625\;X_{40-49y}+0.1784\;X_{60-69y}+0.3613\;X_{70-79y}+0.9356\;X_{\geq 80y}\right)\\ +\;\left(Frequency\;of\;ideal\;dietary\;pattern:\;-0.3791\;X_{4-5/week}-0.1969\;X_{almost\;everyday}\right)\;+\left(Frequency\;of\;eating-out:\;-0.3118\;X_{\leq 1/week}-0.6139\;X_{2-6/week}-1.4118\;X_{\geq 1/day}\right)\\ +\;\left(Breads:\;-0.1349\;X_{\leq 1.0\;slice}-0.1371\;X_{1.1-3.0\;slices}-1.0399\;X_{>3.0\;slices}\right)\;+\;\left(Instant\;noodle:\;-0.6232\;X_{\leq 0.5\;pack}-8.521\;X_{0.6-1.0\;pack}-2.2632\;X_{>1.0\;pack}\right)\;+\;(Fish\\ products-dried:-0.3684\;X_{\leq 1.0\;small\;fish}-0.6865\;X_{1.1-3.0\;small\;fishes}-0.8924\;X_{>3.0\;small\;fish}es)+\;(Fish\;products-paste:\;-0.5585\;X_{\leq 1.0\;piece}-0.8625\;X_{1.1-3.0\;pieces}\\ -0.9547\;X_{>3.0\;pieces})+\;\left(Meat\;products:-0.1959\;X_{\leq 1.0\;piece}-0.8057\;X_{1.1-3.0\;pieces}-1.1846\;X{>}_{3.0\;pieces}\right)\;+\;(Cheese:-0.0092\;X_{\leq 1.0\;slice}-0.0848\;X_{1.1-3.0\;slices}\\ -0.9538\;X_{>3.0\;slices})+\left(Pickles:-0.3947\;X_{\leq 1\;small\;plate}-1.0797\;X_{1.1-3.0\;small\;plates}-2.8844\;X_{>3.0\;small\;plates}\right)-0.8690\;X_{three\;meals\;a\;day} \end{array}$$

$$\begin{array}{l} logit\left(Excess\;intake\right)=2.3600-0.7461\;X_{male}+(Age:\;+0.1054\;X_{20-29y}-0.1381\;X_{30-39y}-0.1432\;X_{40-49y}+0.0222\;X_{60-69y}+0.0801\;X_{70-79y}-0.4693\;X_{\geq 80y})\\ +\left(Frequency\;of\;ideal\;dietary\;pattern:+0.1163\;X_{4-5/week}+0.1592\;X_{almost\;everyday}\right)+(Frequency\;of\;eating-out:\;+0.2275\;X_{\leq 1/week}+0.3717\;X_{2-6/week}+0.4890\;X_{\geq }{}_{1/day})\\ +\left(Breads:\;-0.0759\;X_{\leq 1\;slice}+0.1734\;X_{1.1-3.0\;slices}+1.0399\;X_{>3.0\;slices}\right)+\left(Instant\;noodle:\;+0.1681\;X_{\leq 0.5\;pack}+1.2466\;X_{0.6-1.0\;pack}+2.1970\;X_{>1.0\;pack}\right)\\ +\left(Fish\;products-dried:+0.2086\;X_{\leq 1.0\;small\;fish}+0.7403\;X_{1.1-3.0\;small\;fishes}+0.8546\;X_{>3.0\;small\;fishes}\right)+\left(Fish\;products-paste:+0.1206\;X_{\leq 1.0}{}_{piece}+0.7563\;X_{1.1-3.0\;pieces}+0.9977\;X_{>3.0\;pieces}\right)\\ +\left(Meat\;products:+0.1413\;X_{\leq 1.0\;piece}+0.7078\;X_{1.1-3.0\;pieces}+1.1147\;X_{>3.0\;pieces}\right)+\left(Cheese:+0.0026\;X_{\leq 1.0\;slice}+0.2077\;X_{1.1-3.0\;slices}+0.9723\;X_{>3.0\;slices}\right)\\ +\left(Pickles:+0.4308\;X_{\leq 1.0\;small\;plate}+1.0409\;X_{1.1-3.0\;small\;plates}+1.9499\;X_{>3.0\;small\;plates}\right)+0.7070\;X_{three\;meals\;a\;day,} \end{array}$$

as dummy variable approach respectively.

**Table 3:** Multivariable-adjusted logistic regression analysis of salt intake status in the development group.

		Adequate intake (<7.5 g in men and <6.5 g in women)					Excess intake (≥10 g)				
		β	S.E	Wald	*P*-value	OR (95% CI)	β	S.E	Wald	*P*-value	OR (95% CI)
Sex	Male	-0.0788	0.1010	0.6080	0.44	0.92 (0.76–1.13)	0.7461	0.0838	79.2685	<0.001	2.11 (1.79–2.49)
Age, years	20–29	0.3792	0.2121	3.1947	0.07	1.46 (0.96–2.21)	0.1054	0.1758	0.3593	0.55	1.11 (0.79–1.57)
	30–39	0.5221	0.1898	7.5662	<0.01	1.69 (1.16–2.45)	-0.1381	0.1595	0.7503	0.39	0.87 (0.64–1.19)
	40–49	0.2625	0.1779	2.1756	0.14	1.30 (0.92–1.84)	-0.1432	0.1430	1.0023	0.32	0.87 (0.65–1.15)
	50–59	Ref.					Ref.				
	60–69	0.1784	0.1766	1.0206	0.31	1.20 (0.85–1.69)	0.0222	0.1335	0.0277	0.87	1.02 (0.79–1.33)
	70–79	0.3613	0.1868	3.7397	0.05	1.44 (1.00–2.07)	0.0801	0.1448	0.3056	0.58	1.08 (0.82–1.44)
	≥80	0.9356	0.2047	20.8810	<0.001	2.55 (1.71–3.81)	-0.4693	0.1822	6.6329	<0.05	0.63 (0.44–0.89)
Frequency of ideal dietary pattern	≤3 days a week	Ref.					Ref.				
	4–5 days a week	-0.3791	0.1130	11.2604	<0.001	0.68 (0.55–0.85)	0.1163	0.0961	1.4659	0.23	1.12 (0.93–1.36)
	Almost everyday	-0.1969	0.1387	2.0158	0.16	0.82 (0.63–1.08)	0.1592	0.1205	1.7456	0.19	1.17 (0.93–1.48)
Frequency of eating-out	Never	Ref.					Ref.				
	Once a week or less	-0.3118	0.1228	6.4502	<0.05	0.73 (0.58–0.93)	0.2275	0.1054	4.6630	<0.05	1.26 (1.02–1.54)
	2–6 times a week	-0.6139	0.1786	11.8204	<0.001	0.54 (0.38–0.77)	0.3717	0.1476	6.3461	<0.05	1.45 (1.09–1.94)
	Once a day or more	-1.4118	0.3685	14.6747	<0.001	0.24 (0.12–0.50)	0.4890	0.2373	4.2476	<0.05	1.63 (1.02–2.60)
Breads	None (0 g)	Ref.					Ref.				
	≤1.0 slice (1–60 g)	-0.1349	0.1270	1.1281	0.29	0.87 (0.68–1.12)	-0.0759	0.1048	0.5240	0.47	0.93 (0.75–1.14)
	1.1–3.0 slices (61–180 g)	-0.1371	0.1285	1.1374	0.29	0.87 (0.68–1.12)	0.1734	0.1042	2.7705	0.10	1.19 (0.97–1.46)
	> 3.0 slices (> 180 g)	-1.0399	0.7672	1.8372	0.18	0.35 (0.08–1.59)	1.0039	0.4626	4.7085	<0.05	2.73 (1.10–6.76)
Instant noodle	None (0 g)	Ref.					Ref.				
	≤0.5 pack (1–45 g)	-0.6232	0.6775	0.8461	0.36	0.54 (0.14–2.02)	0.1681	0.5679	0.0876	0.77	1.18 (0.39–3.60)
	0.6–1.0 pack (46–90 g)	-0.8521	0.3612	5.5656	<0.05	0.43 (0.21–0.87)	1.2466	0.2689	21.4860	<0.001	3.48 (2.05–5.89)
	> 1.0 pack (> 90 g)	-2.2632	0.6078	13.8643	<0.001	0.10 (0.03–0.34)	2.1970	0.3426	41.1341	<0.001	9.00 (4.60–17.6)
Fish products -salted, half-dried, and dried	None (0 g)	Ref.					Ref.				
	≤1.0 small fish (1–20 g)	-0.3684	0.1228	9.0005	<0.01	0.69 (0.54–0.88)	0.2086	0.1001	4.3433	<0.05	1.23 (1.01–1.50)
	1.1–3.0 small fishes (21–60 g)	-0.6865	0.1808	14.4155	<0.001	0.50 (0.35–0.72)	0.7403	0.1334	30.8013	<0.001	2.10 (1.61–2.72)
	> 3.0 small fishes (> 60 g)	-0.8924	0.1925	21.4947	<0.001	0.41 (0.28–0.60)	0.8546	0.1358	39.5752	<0.001	2.35 (1.80–3.07)
Fish products -fish paste	None (0 g)	Ref.					Ref.				
	≤1.0 piece (1–15 g)	-0.5585	0.1913	8.5263	<0.01	0.57 (0.39–0.83)	0.1206	0.1432	0.7091	0.40	1.13 (0.85–1.49)
	1.1–3.0 pieces (16–45 g)	-0.8625	0.2042	17.8366	<0.001	0.42 (0.28–0.63)	0.7563	0.1357	31.0780	<0.001	2.13 (1.63–2.78)
	> 3.0 pieces (> 45 g)	-0.9547	0.2530	14.2435	<0.001	0.38 (0.23–0.63)	0.9977	0.1721	33.6007	<0.001	2.71 (1.94–3.80)
Meat products -ham and sausage	None (0 g)	Ref.					Ref.				
	≤1.0 piece (1–20 g)	-0.1959	0.1227	2.5496	0.11	0.82 (0.65–1.05)	0.1413	0.1037	1.8572	0.17	1.15 (0.94–1.41)
	1.1–3.0 pieces (21–60 g)	-0.8057	0.1585	25.8540	<0.001	0.45 (0.33–0.61)	0.7078	0.1138	38.6514	<0.001	2.03 (1.62–2.54)
	> 3.0 pieces (> 60 g)	-1.1846	0.3682	10.3495	<0.01	0.31 (0.15–0.63)	1.1147	0.2350	22.5060	<0.001	3.05 (1.92–4.83)
Cheese	None (0 g)	Ref.					Ref.				
	≤1.0 slice (1–15 g)	-0.0092	0.1819	0.0025	0.96	0.99 (0.69–1.42)	0.0026	0.1457	0.0003	0.99	1.00 (0.75–1.33)
	1.1–3.0 slices (16–45 g)	-0.0848	0.1788	0.2251	0.64	0.92 (0.65–1.30)	0.2077	0.1442	2.0753	0.15	1.23 (0.93–1.63)
	> 3.0 slices (> 45 g)	-0.9538	0.7823	1.4867	0.22	0.39 (0.08–1.78)	0.9723	0.5000	3.7817	0.05	2.64 (0.99–7.05)
Pickles	None (0 g)	Ref.					Ref.				
	≤1.0 small plate (1–20 g)	-0.3947	0.1190	11.0105	<0.001	0.67 (0.53–0.85)	0.4308	0.0958	20.2159	<0.001	1.54 (1.28–1.86)
	1.1–3.0 small plates (21–60 g)	-1.0797	0.1988	29.5003	<0.001	0.34 (0.23–0.50)	1.0409	0.1318	62.3505	<0.001	2.83 (2.19–3.67)
	> 3.0 small plates (> 60 g)	-2.8844	0.7214	15.9867	<0.001	0.06 (0.01–0.23)	1.9499	0.2569	57.6129	<0.001	7.03 (4.25–11.6)

OR, odds ratio; CI, confidence interval; S.E., standard error; Ref., reference.

Multivariable-adjusted model included three meals other than the variables in the table (β, -0.8690; *P*-value, <0.001 for adequate intake and β, 0.7070; *P*-value, <0.001 for excess intake).

Intercepts were 0.4692 for adequate intake and -2.3600 for excess intake.

Table 4 shows the predictive performance of the final model in the development and validation groups. The AUCs were 0.747 (95% confidence interval (CI) = 0.726–0.767) for sex-specific DRI-J and 0.741 (95% CI = 0.723–758) for above-average intake in the development group. The corresponding values in the validation group were 0.734 (0.713–0.754) and 0.730 (0.713–0.748), respectively. The model performance for sensitivity and specificity for different probability cut-offs is shown in Table 5. For identifying adequate intake, the highest AUC was 0.6848 for the prediction model when applying a cut-off of 0.3. For identifying excess intake, the corresponding value was 0.6866 when applying a cut-off of 0.8.

**Table 4:** Performance comparison for prediction in the development and validation groups.

	Development group	Validation group
Salt intake < sex-specific DRI		
Sensitivity (95% CI)	0.718 (0.708–0.729)	0.707 (0.696–0.718)
Specificity (95% CI)	0.525 (0.514–0.537)	0.524 (0.513–0.536)
AUC (95% CI)	0.747 (0.726–0.767)	0.734 (0.713–0.754)
Salt intake ≥ 10 g		
Sensitivity (95% CI)	0.608 (0.597–0.619)	0.603 (0.592–0.614)
Specificity (95% CI)	0.635 (0.623–0.646)	0.630 (0.618–0.641)
AUC (95% CI)	0.741 (0.723–0.758)	0.730 (0.713–0.748)

CI, confidence interval; AUC, areas under receiver operating characteristic curve.

**Table 5:** Model performance in the development and validation groups at different cut-off probabilities.

	Cut-off point							
	>0.2	>0.3	>0.4	>0.5	>0.6	>0.7	>0.8	>0.9
**Development group**								
Salt intake < sex-specific DRI								
Sensitivity	0.9292	0.8817	0.8331	0.7925	0.765	0.7428	0.7272	0.7169
Specificity	0.1902	0.2415	0.2707	0.2921	0.305	0.3128	0.3179	0.3213
AUC	0.6792	0.6848	0.6557	0.6269	0.6051	0.5835	0.5676	0.5573
Salt intake ≥ 10 g								
Sensitivity	0.9940	0.9745	0.9588	0.9393	0.9212	0.9037	0.8890	0.8769
Specificity	0.0283	0.0799	0.1226	0.1553	0.1888	0.2173	0.2354	0.2474
AUC	0.5334	0.5816	0.6221	0.6419	0.6651	0.6814	0.6866	0.6865
**Validation group**								
Salt intake < sex-specific DRI								
Sensitivity	0.9240	0.8634	0.8227	0.7928	0.7645	0.7496	0.7312	0.7173
Specificity	0.1861	0.2404	0.2708	0.2914	0.3067	0.3145	0.3199	0.3215
AUC	0.6652	0.6557	0.6401	0.6264	0.6068	0.5962	0.5768	0.5583
Salt intake ≥ 10 g								
Sensitivity	0.9948	0.9754	0.959	0.9448	0.9217	0.9023	0.8852	0.8693
Specificity	0.0304	0.0855	0.1256	0.1582	0.1847	0.2051	0.2269	0.2413
AUC	0.5377	0.5913	0.6269	0.6545	0.6597	0.6611	0.6681	0.6658

DRI, dietary reference intakes; AUC, areas under receiver operating characteristic curve.

When re-analysed using 7.0 g the median value of sex-specific DRI (salt intake) for adequate intake, the results were not substantially altered: the AUCs in the development and validation groups were 0.763 (0.744–0.782) and 0.752 (0.733–0.772), respectively, and the prediction model had the highest AUC when applying a cut-off of 0.3 (S2–S4 Tables).

## Discussion

From the NHNS data, based on the utilisation of nutrition labels, the frequency of eating-out, and the ideal combination of dishes, it was observed that the level of sodium intake differed significantly from the DRI-J 2020 and average intakes. Furthermore, from the brief questionnaire including three dietary behaviours and seven food consumptions, it was noticed that the developed method helps to categorise the sodium intake as adequate (less than the DRI-J) intake and excess (more than the average) intake, respectively.

We observed that the proportion of individuals with higher frequency of eating-out were lower among those with adequate intake, and higher among those with excess intake than those with lower frequency. It was reported that the degree of sodium intake varied greatly with the frequency of eating-out as restaurant food contains higher concentrations of sodium [12, 13]. Some restaurants in Japan actively provide a low salt menu and display nutrients, mainly salt equivalent, which is also encouraged by the national and local governments, however, its impact is uncertain. This dietary environment potentially supports our results of low sodium intake among those who use nutrition labels even while eating out, however, the dissemination of more easy-to-understand nutrition labels is required for raising awareness. We expect that this screening method will encourage recognition of salt intake.

Additionally, food labelling with respect to nutritional content is regulated by the Food Labelling Act, effective from April 1, 2015 (formerly regulated by the Health Promotion Act). Due to the change in the regulation, nutrition labelling has been made mandatory and sodium has been replaced with salt equivalent, making it easier to compare with the salt equivalent values shown in DRI-J [14, 15]. We hope that the reduction in sodium intake will therefore progress due to the synergy of this awareness-raising method and the dietary environment of nutrition labelling.

However, instead of the utilisation of nutrition labels, an ideal combination of dishes played a key role in the final prediction model for sodium intake defined by sex-specific DRI-J and above-average intake. An ideal combination of dishes containing the staple food (i.e., rice, breads, and noodles), a main dish (i.e., dishes with fishes, meats, eggs, and soybeans), and a side dish (i.e., dishes with vegetables, seaweeds, and mushrooms) has been recommended for good nutritional balance in the Japanese diet culture. Although side dishes such as boiled food and stir-fried vegetables increase salt content, they act as a source of dietary fibre and some vitamins and minerals.

We found that the correlation between portion sizes of processed food and sodium intake varied greatly, depending on the kind of processed food consumed. In previous studies, some affected the sodium intake significantly even in small amounts, and some affected it only in larger quantities [16–20]. These results may effectively represent the trends in the Japanese food market and the sources of salt intake.

The main strength of this study was its representativeness of the Japan population, as eating behaviour, including factors of dietary environment, were evaluated using the NHNS data. A further strength was that we set several easy-to-understand quantitative units and carefully analysed them by combining quality and quantity. This study, however, had several limitations. First, the sodium intake may possibly be affected by other dietary behaviours, which was not assessed in the questionnaire. However, our screening method using the brief question might be helpful for people including those who are not interested in the nutritional composition of foods. Second, although sodium intake can be estimated from urine [21, 22], it was only compared to estimates from a dietary survey, as the data did not include urine data for biochemically validation. Third, due to the limitation in the sample size, multiple comparisons using other behaviours and narrow ranges of sodium intake were not possible. Forth, the developed method had lower specificity but high sensitivity. However, it was useful to identify the criteria that would lead to high or adequate intake. Finally, although the developed method may be able to assess sodium intake, it is unclear whether it will actually lead to the consolidation of knowledge and eventually to behavioural changes. Therefore, verification of the effectiveness of this method is required in future studies.

In conclusion, this study provided evidence of the association between dietary habits and sodium intake, and a method to easily measure sodium intake from dietary habits in Japanese. We expect this method to help disseminate the reference values of sodium intake based on DRI-J 2020 for health and nutrition education, and to promote behavioural change towards reducing sodium intake in many people, including those with little knowledge about nutrition.

## Supporting information

S1 Table Comparison of dietary behaviours according to sodium intakes in the population without skipping behaviours. (DOCX)

S2 Table Multivariable-adjusted logistic regression analysis of the factors of sodium intake in the development group. (DOCX)

S3 Table Performance comparison for prediction in the development and validation groups. (DOCX)

S4 Table Model performance in the development and validation groups at different cut-off probabilities. (DOCX)

## References

[1] Ministry of Health, Labour and Welfare of Japan. The dietary reference intakes for Japanese, 2020 [cited 2019 Sep 27]. https://www.mhlw.go.jp/stf/shingi/other-kenkou_539644.html (in Japanese).

[2] Ministry of Health, Labour and Welfare of Japan. Dietary Reference Intakes for Japanese (2015). 2015 [cited 2019 Sep 27]. https://www.mhlw.go.jp/stf/seisakunitsuite/bunya/0000208970.html (in Japanese)

[3] Powles J, Fahimi S, Micha R, Khatibzadeh S, Shi PL, Ezzati M, et al Global, regional and national sodium intakes in 1990 and 2010: a systematic analysis of 24 h urinary sodium excretion and dietary surveys worldwide. BMJ Open. 2013;3. 10.1136/bmjopen-2013-003733 PMC3884590 24366578

[4] WHO. Guideline: Sodium intake for adults and children [cited 2019 Sep 27]. https://www.who.int/nutrition/publications/guidelines/sodium_intake_printversion.pdf 23658998

[5] Ministry of Health, Labour and Welfare. Annual Report of the National Health and Nutrition Survey in Japan, 2016 [cited 2019 Sep 27]. https://www.mhlw.go.jp/bunya/kenkou/eiyou/h28-houkoku.html (in Japanese)

[6] Wang CJ, Li YQ, Wang L, Li LL, Guo YR, Zhang LY, et al Development and evaluation of a simple and effective prediction approach for identifying those at high risk of dyslipidemia in rural adult residents. PLoS One. 2012;7:e43834 10.1371/journal.pone.0043834 22952780 PMC3429495

[7] Wijnhoven HAH, Elstgeest LEM, de Vet HCW, Nicolaou M, Snijder MB and Visser M. Development and validation of a short food questionnaire to screen for low protein intake in community-dwelling older adults: The Protein Screener 55+ (Pro55+). PLoS One. 2018;13:e0196406 10.1371/journal.pone.0196406 29791454 PMC5965846

[8] Martinez FJ, Raczek AE, Seifer FD, Conoscenti CS, Curtice TG, D’Eletto T, et al Development and initial validation of a self-scored COPD Population Screener Questionnaire (COPD-PS). COPD. 2008;5:85–95. 10.1080/15412550801940721 18415807 PMC2430173

[9] Okada E, Takahashi K, Takimoto H, Takabayashi S, Kishi T, Kobayashi T, et al Dietary patterns among Japanese adults: findings from the National Health and Nutrition Survey, 2012. Asia Pac J Clin Nutr. 2018;27:1120–1130. 10.6133/apjcn.042018.06 30272860

[10] Council for Science and Technology, Ministry of Education, Culture, Sports, Science and Technology, the Government of Japan. Standard Tables of Food Composition in Japan, 2010. Tokyo: Official Gazette Cooperation of Japan; 2010.

[11] Hollander M, Chicken E and Wolfe DA. Nonparametric statistical methods. 3rd ed New York: John Wiley & Sons Inc 2014.

[12] Brown IJ, Tzoulaki I, Candeias V and Elliott P. Salt intakes around the world: implications for public health. Int J Epidemiol. 2009;38:791–813. 10.1093/ije/dyp139 19351697

[13] Guthrie JF, Lin BH and Frazao E. Role of food prepared away from home in the American diet, 1977–78 versus 1994–96: Changes and consequences. J Nutr Educ Behav. 2002;34:140–150. 10.1016/s1499-4046(06)60083-3 12047838

[14] Okuda N, Nishi N, Ishikawa-Takata K, Yoshimura E, Horie S, Nakanishi T, et al Understanding of sodium content labeled on food packages by Japanese people. Hypertens Res. 2014;37:467–471. 10.1038/hr.2013.149 24173359

[15] Watanabe S, Melby M and Aiba N. Food safety and food labeling from the viewpoint of the consumers. Asia Pac J Clin Nutr. 2009;18:532–537. 19965344

[16] Cappuccio FP. Salt and cardiovascular disease. BMJ. 2007;334:859–860. 10.1136/bmj.39175.364954.BE 17463420 PMC1857801

[17] Trieu K, Neal B, Hawkes C et al Salt Reduction Initiatives around the World—A Systematic Review of Progress towards the Global Target. PLoS One. 2015;10:e0130247 10.1371/journal.pone.0130247 26201031 PMC4511674

[18] Campbell N, Legowski B, Legetic B, Ferrante D, Nilson E, Campbell C, et al Targets and Timelines for Reducing Salt in Processed Food in the Americas. J Clin Hypertens. 2014;16:619–623. 10.1111/jch.12379 PMC8031643 25077666

[19] Trevena H, Thow AM, Dunford E, Wu JHY and Neal B. Protocol for a cluster-randomised trial to determine the effects of advocacy actions on the salt content of processed foods. BMC Public Health. 2016;16:75 10.1186/s12889-016-2743-4 26809561 PMC4727283

[20] Nghiem N, Blakely T, Cobiac LJ, Cleghorn CL and Wilson N. The health gains and cost savings of dietary salt reduction interventions, with equity and age distributional aspects. BMC Public Health. 2016;16:423 10.1186/s12889-016-3102-1 27216490 PMC4877955

[21] Powles J, Fahimi S, Micha R, Khatibzadeh S, Shi P, Ezzati M, et al; Global Burden of Diseases Nutrition and Chronic Diseases Expert Group (NutriCoDE). Global, regional and national sodium intakes in 1990 and 2010: a systematic analysis of 24 h urinary sodium excretion and dietary surveys worldwide. BMJ Open 2013;3:e003733 10.1136/bmjopen-2013-003733 24366578 PMC3884590

[22] Mozaffarian D Fahimi S Singh GM, Micha R, Khatibzadeh S, Engell RE, et al; the Global Burden of Diseases Nutrition and Chronic Diseases Expert Group. Global sodium consumption and death from cardiovascular causes. N Engl J Med. 2014;371:624–634. 10.1056/NEJMoa1304127 25119608

