# Supplementary material for: Development of a screening method for determining sodium intake based on the Dietary Reference Intakes for Japanese, 2020: A cross-sectional analysis of the National Health and Nutrition Survey, Japan
Source: PLoS One. 2020 Sep 15;15(9):e0235749. doi: 10.1371/journal.pone.0235749 (PMC7491721; doi:10.1371/journal.pone.0235749)
Supplement: S1 Table — (DOCX) [file pone.0235749.s001.docx]

**S1 Table**. Comparison of dietary behaviours according to sodium intakes in the population without skipping behaviours

|  | Sodium (salt equivalent) intake | | | | | | | |
| --- | --- | --- | --- | --- | --- | --- | --- | --- |
|  | < 6.5 g | | 6.5-<7.5 g | | 7.5-<10 g | | ≥10 g | |
| **All** |  |  |  |  |  |  |  |  |
| Utilisation of Nutrition label | 44.1 |  | 48.8 | ^‡^ | 44.2 | ^*^ | 39.3 | ^‡,*^ |
| Needs of nutrition label |  |  |  |  |  |  |  |  |
| Carbohydrate | 17.6 | ^*^ | 17.6 |  | 14.7 |  | 13.8 | ^*^ |
| Sodium (salt equivalent) | 30.7 | ^*^ | 29.1 |  | 28.3 |  | 25.6 | ^*^ |
| Frequency of eating-out, ≥2 /week | 8.8 | ^‡,†,‡’^ | 16.4 | ^‡^ | 13.7 | ^†,*^ | 17.3 | ^‡’,*^ |
| Frequency of ideal combination of dishes, ≥4 /week | 67.3 | ^*,†^ | 74.2 | * | 71.9 |  | 74.2 | ^†^ |
| **Low-frequency of eating-out**, **<2 /week** |  |  |  |  |  |  |  |  |
| Utilisation of Nutrition label | 44.5 |  | 47.4 | ^*^ | 43.8 |  | 40.3 | ^*^ |
| Needs of nutrition label |  |  |  |  |  |  |  |  |
| Carbohydrate | 16.9 |  | 18.1 |  | 14.4 |  | 13.9 |  |
| Sodium (salt equivalent) | 32.7 | ^*^ | 29.8 |  | 29.4 |  | 26.1 | ^*^ |
| Frequency of ideal combination of dishes, ≥4 /week | 68.8 | ^*,‡^ | 76.6 | ^*^ | 72.3 | ^*’^ | 77.1 | ^‡,*’^ |
| **High-frequency of eating-out,** **≥2 /week** |  |  |  |  |  |  |  |  |
| Utilisation of Nutrition label | 40.3 |  | 56.1 | ^†^ | 46.7 | ^*^ | 34.3 | ^†,*^ |
| Needs of nutrition label |  |  |  |  |  |  |  |  |
| Carbohydrate | 25.4 |  | 14.6 |  | 16.4 |  | 13.5 |  |
| Sodium (salt equivalent) | 10.4 |  | 25.6 |  | 21.5 |  | 22.8 |  |
| Frequency of ideal combination of dishes, ≥4 /week | 50.0 | ^*^ | 62.2 |  | 68.7 | ^*^ | 60.5 |  |

Variables with the same superscript are significantly different, as determined using the multiple comparison test (<0.001, ^‡^; <0.01, ^†^; <0.05, ^*^).

An ideal combination of dishes refers to meals with staple food, main dish, and side dish consumed more than twice a day.

Note: Missing values were excluded in the analysis for each item.
