# Supplementary material for: Development of a screening method for determining sodium intake based on the Dietary Reference Intakes for Japanese, 2020: A cross-sectional analysis of the National Health and Nutrition Survey, Japan
Source: PLoS One. 2020 Sep 15;15(9):e0235749. doi: 10.1371/journal.pone.0235749 (PMC7491721; doi:10.1371/journal.pone.0235749)
Supplement: S2 Table — (DOCX) [file pone.0235749.s002.docx]

**S2 Table**.　 Multivariable-adjusted logistic regression analysis of the factors of sodium intake in the development group

|  |  | Adequate intake (<7.0 g in men and women) | | | | |
| --- | --- | --- | --- | --- | --- | --- |
|  |  | β | S.E | Wald | *P*-value | OR (95%CI) |
| Sex | Male | -0.8073 | 0.1039 | 60.3485 | <0.001 | 0.45 (0.36-0.55) |
| Age, years | 20-29 | 0.2166 | 0.2082 | 1.0825 | 0.30 | 1.24 (0.83-1.87) |
|  | 30-39 | 0.3708 | 0.1849 | 4.0217 | <0.05 | 1.45 (1.01-2.08) |
|  | 40-49 | 0.3357 | 0.1685 | 3.9705 | <0.05 | 1.40 (1.01-1.95) |
|  | 50-59 | Ref. |  |  |  |  |
|  | 60-69 | -0.0909 | 0.1712 | 0.2819 | 0.60 | 0.91 (0.65-1.28) |
|  | 70-79 | 0.1378 | 0.1809 | 0.5807 | 0.45 | 1.15 (0.81-1.64) |
|  | ≥80 | 0.7237 | 0.1997 | 13.1305 | <0.001 | 2.06 (1.39-3.05) |
| Frequency of ideal dietary pattern | ≤3 days a week | Ref. |  |  |  |  |
|  | 4-5 days a week | -0.2792 | 0.1121 | 6.2049 | <0.05 | 0.76 (0.61-0.94) |
|  | Almost everyday | -0.1620 | 0.1384 | 1.3703 | 0.24 | 0.85 (0.65-1.12) |
| Frequency of eating-out | Never | Ref. |  |  |  |  |
|  | Once a week or less | -0.3595 | 0.1215 | 8.7524 | <0.01 | 0.70 (0.55-0.89) |
|  | 2-6 times a week | -0.6981 | 0.1808 | 14.9092 | <0.001 | 0.50 (0.35-0.71) |
|  | Once a day or more | -1.2402 | 0.3550 | 12.2022 | <0.001 | 0.29 (0.14-0.58) |
| Breads | None (0 g) | Ref. |  |  |  |  |
|  | ≤1.0 slice (1-60 g) | -0.1014 | 0.1240 | 0.6685 | 0.41 | 0.90 (0.71-1.15) |
|  | 1.1-3.0 slices (61-180 g) | -0.1162 | 0.1269 | 0.8384 | 0.36 | 0.89 (0.69-1.14) |
|  | > 3.0 slices (> 180 g) | -0.6622 | 0.6622 | 1.0001 | 0.32 | 0.52 (0.14-1.89) |
| Instant noodle | None (0 g) | Ref. |  |  |  |  |
|  | ≤0.5 pack (1-45 g) | -0.4219 | 0.6306 | 0.4477 | 0.50 | 0.66 (0.19-2.26) |
|  | 0.6-1.0 pack (46-90 g) | -1.2711 | 0.4160 | 9.3359 | <0.01 | 0.28 (0.12-0.63) |
|  | > 1.0 pack (> 90 g) | -2.6751 | 0.7366 | 13.1887 | <0.001 | 0.07 (0.02-0.29) |
| Fish products: salted, half-dried, and dried | None (0 g) | Ref. |  |  |  |  |
|  | ≤1.0 small fish (1-20 g) | -0.2414 | 0.1188 | 4.1275 | <0.05 | 0.79 (0.62-0.99) |
|  | 1.1-3.0 small fishes (21-60 g) | -0.7142 | 0.1772 | 16.2409 | <0.001 | 0.49 (0.35-0.69) |
|  | > 3.0 small fishes (> 60 g) | -0.9586 | 0.1952 | 24.1120 | <0.001 | 0.38 (0.26-0.56) |
| Fish products: fish paste | None (0 g) | Ref. |  |  |  |  |
|  | ≤1.0 piece (1-15 g) | -0.6934 | 0.1927 | 12.9398 | <0.001 | 0.50 (0.34-0.73) |
|  | 1.1-3.0 pieces (16-45 g) | -0.9704 | 0.2023 | 23.0178 | <0.001 | 0.38 (0.25-0.56) |
|  | > 3.0 pieces (> 45 g) | -0.9532 | 0.2439 | 15.2678 | <0.001 | 0.39 (0.24-0.62) |
| Meat products: ham and sausage | None (0 g) | Ref. |  |  |  |  |
|  | ≤1.0 piece (1-20 g) | -0.1320 | 0.1196 | 1.2176 | 0.27 | 0.88 (0.69-1.11) |
|  | 1.1-3.0 pieces (21-60 g) | -0.8218 | 0.1560 | 27.7515 | <0.001 | 0.44 (0.32-0.60) |
|  | > 3.0 pieces (> 60 g) | -1.2031 | 0.3712 | 10.5030 | <0.01 | 0.30 (0.15-0.62) |
| Cheese | None (0 g) | Ref. |  |  |  |  |
|  | ≤1.0 slice (1-15 g) | -0.1729 | 0.1827 | 0.8959 | 0.34 | 0.84 (0.59-1.20) |
|  | 1.1-3.0 slices (16-45 g) | -0.1393 | 0.1736 | 0.6434 | 0.42 | 0.87 (0.62-1.22) |
|  | > 3.0 slices (> 45 g) | -0.9148 | 0.7765 | 1.3880 | 0.24 | 0.40 (0.09-1.84) |
| Pickles | None (0 g) | Ref. |  |  |  |  |
|  | ≤1.0 small plate (1-20 g) | -0.3918 | 0.1174 | 11.1379 | <0.001 | 0.68 (0.54-0.85) |
|  | 1.1-3.0 small plates (21-60 g) | -0.9855 | 0.1883 | 27.4041 | <0.001 | 0.37 (0.26-0.54) |
|  | > 3.0 small plates (> 60 g) | -3.6510 | 1.0112 | 13.0369 | <0.001 | 0.03 (0.004-0.19) |

OR, odds ratio; CI, confidence interval; S.E., standard error; Ref., reference.

Multivariable-adjusted model included three meals other than the variables in the table (β, -0.8577; *P*-value, <0.001).

Intercepts were 0.9571.
