# Supplementary material for: Development of a screening method for determining sodium intake based on the Dietary Reference Intakes for Japanese, 2020: A cross-sectional analysis of the National Health and Nutrition Survey, Japan
Source: PLoS One. 2020 Sep 15;15(9):e0235749. doi: 10.1371/journal.pone.0235749 (PMC7491721; doi:10.1371/journal.pone.0235749)
Supplement: S3 Table — (DOCX) [file pone.0235749.s003.docx]

**S3 Table.** Performance comparison for prediction in the development and validation groups

|  | Development group | Validation group |
| --- | --- | --- |
| **Salt intake < 7.0g** |  |  |
| Sensitivity (95% CI) | 0.728 (0.717-0.739) | 0.718 (0.707-0.728) |
| Specificity (95% CI) | 0.532 (0.520-0.543) | 0.532 (0.521-0.544) |
| AUC (95% CI) | 0.763 (0.744-0.782) | 0.752 (0.733-0.772) |
| CI, confidence interval; AUC, areas under receiver operating characteristic curve. | | |
