# Supplementary material for: Development of a screening method for determining sodium intake based on the Dietary Reference Intakes for Japanese, 2020: A cross-sectional analysis of the National Health and Nutrition Survey, Japan
Source: PLoS One. 2020 Sep 15;15(9):e0235749. doi: 10.1371/journal.pone.0235749 (PMC7491721; doi:10.1371/journal.pone.0235749)
Supplement: S4 Table — (DOCX) [file pone.0235749.s004.docx]

**S4 Table.** Model performance in the development and validation groups at different cut-off probabilities

|  | Cut-off point | | | | | | | |
| --- | --- | --- | --- | --- | --- | --- | --- | --- |
|  | >0.2 | >0.3 | >0.4 | >0.5 | >0.6 | >0.7 | >0.8 | >0.9 |
| **Salt intake <7.0 g** |  |  |  |  |  |  |  |  |
| **Development group** |  |  |  |  |  |  |  |  |
| Sensitivity | 0.9439 | 0.9043 | 0.8627 | 0.8195 | 0.793 | 0.7699 | 0.7549 | 0.7373 |
| Specificity | 0.1804 | 0.2284 | 0.2613 | 0.2794 | 0.2932 | 0.3045 | 0.3114 | 0.3177 |
| AUC | 0.6864 | 0.6989 | 0.6859 | 0.6484 | 0.6292 | 0.6116 | 0.5995 | 0.5826 |
| **Validation group** |  |  |  |  |  |  |  |  |
| Sensitivity | 0.9391 | 0.8898 | 0.8489 | 0.8219 | 0.7903 | 0.7717 | 0.7550 | 0.7373 |
| Specificity | 0.1848 | 0.2314 | 0.2585 | 0.2787 | 0.2928 | 0.3028 | 0.3107 | 0.3179 |
| AUC | 0.6858 | 0.6819 | 0.6611 | 0.6509 | 0.6247 | 0.6118 | 0.5985 | 0.5829 |
| DRI, dietary reference intakes; AUC, areas under receiver operating characteristic curve. | | | | | | | | |
